# Mechanisms linking bariatric surgery to adipose tissue, glucose metabolism, fatty liver disease and gut microbiota

**DOI:** 10.1007/s00423-023-02821-8

**Published:** 2023-02-24

**Authors:** Saverio Latteri, Maria Sofia, Stefano Puleo, Angelica Di Vincenzo, Saverio Cinti, Sergio Castorina

**Affiliations:** 1 Department of Medical, Surgical Sciences and Advanced Technologies “G.F. Ingrassia”, University of Catania, Catania, Italy; 2 Department of General Surgery, Cannizzaro Hospital, Via Messina 829, 95126 Catania, Italy; 3 Mediterranean Foundation “GB Morgagni”, Catania, Italy; 4 Department of Experimental and Clinical Medicine, Center of Obesity, Marche Polytechnic University, Via Tronto 10A, 60020 Ancona, Italy

**Keywords:** Bariatric surgery, Diabetes, Adipose tissue, Gut microbiota, Non-alcoholic fatty liver disease

## Abstract

**Purpose:**

In the last 20 years, bariatric surgery has achieved an important role in translational and clinical research because of obesity comorbidities. Initially, a tool to lose weight, bariatric surgery now has been shown to be involved in several metabolic pathways.

**Methods:**

We conducted a narrative review discussing the underlying mechanisms that could explain the impact of bariatric surgery and the relationship between obesity and adipose tissue, T2D, gut microbiota, and NAFLD.

**Results:**

Bariatric surgery has an impact in the relation between obesity and type 2 diabetes, but in addition  it induces the white-to-brown adipocyte trans-differentiation, by enhancing thermogenesis. Another issue is the connection of bariatric surgery with the gut microbiota and its role in the complex mechanism underlying weight gain.

**Conclusion:**

Bariatric surgery modifies gut microbiota, and these modifications influence lipid metabolism, leading to improvement of non-alcoholic fatty liver disease.

## Introduction

Obesity is a major public health problem that has been increasing worldwide [1]. This multifactorial disease is associated with an increased risk of developing several medical conditions, such as insulin resistance, hypertension, dyslipidaemia, non-alcoholic fatty liver disease (NAFLD), cardiovascular disease and even some types of cancers. In addition, it is the major risk factor for type 2 diabetes (T2D) [2, 3].

Bariatric surgery has been demonstrated to successfully achieve significant and sustainable weight loss and improvement of associated comorbidities [4, 5]. The benefits observed in metabolic disease, independently of weight loss, define bariatric surgery as metabolic surgery. In 1978, L. Varco described metabolic surgery as “the operative manipulation of a normal organ system to achieve a biological result for a potential health gain” [6]. The first surgical management for obesity was in 1952, when V. Henrikson, a Swedish surgeon, performed a 105-cm small bowel resection on a woman with obesity [7]. Since then, there have been six historically dominant procedures in bariatric surgery [6]: jejunoileal by-pass (JIB), Roux-en-Y gastric by-pass (RYGB), then modified in one anastomosis gastric by-pass (OAGB), vertical banded gastroplasty (VBG), biliopancreatic diversion (BPD) and its modification duodenal switch (DS), adjustable gastric banding (AGB) and sleeve gastrectomy (SG) (Table 1). Globally, the number of surgical procedures has dramatically increased from 146,301 procedures carried out in 2003 to 604,223 surgical procedures in 2018 [8, 9]. SG is the most performed bariatric procedure (55.4%), followed by RYGB (29.3%), then OAGB (6.6%), whereas no other single surgical procedure exceeds 1.5% [8].

**Table 1:** History of bariatric surgery

Year	Surgeon	Procedure
1954	Payne	Jejunoileal by-pass
1966	Mason	Gastric by-pass
1973	Printen	Gastroplasty
1977	Griffen	Roux-en-Y gastric by-pass
1978	Wilkinson	Nonadjustable gastric band
1979	Scopinaro	Biliopancreatic diversion
1982	Mason	Vertical banded gastroplasty
1985	Garren-Edwards	First endoscopic endoluminal gastric balloon
1986	Kuzmak	Adjustable gastric band
1988	Hess	BPD with duodenal switch
1991	Apollo*	Bioenteric intragastric balloon
1993	Forsell	Laparoscopic adjustable gastric band
1994	Hess	Laparoscopic VBG
1994	Wittgrove	Laparoscopic RYGB
1997	Rutledge	One anastomosis gastric by-pass
2003	Regan	Laparoscopic sleeve gastrectomy
2012	Thompson	Endoscopic sleeve gastroplasty
2013	Espinos	Primary obesity surgery endoluminal (POSE)

^*^Apollo Endosurgery, Austin, TX, USA

*BPD*, biliopancreatic diversion; *VBG*, vertical banded gastroplasty; *RYGB*, Roux-en-Y gastric by-pass

These procedures were originally designed to achieve weight loss, but now it is known that bariatric surgery involves molecular, anatomical and physiological alterations even by weight-independent mechanisms, with long-term effects [4, 5].

Anatomical changes resulting from metabolic surgery can alter physiology. Anatomical modifications in SG include excision of the enteroendocrine cells (EECs) bearing greater curvature of the stomach, whereas RYGB anatomical rearrangements decrease the time required to the nutrients for transit into the small bowel, by-passing the stomach, duodenum and early jejunum. These anatomical changes induce weight loss but might have additional consequences, that are weight-independent, with benefits on obesity comorbidities [10, 11] (Table 2).

**Table 2:** Obesity-associated diseases and their improvement after bariatric surgery

Type 2 diabetes mellitus	• Haemoglobin A_1c_ < 6.0–7.0% • Absence of medication • Fasting glucose < 100 mg/dl • Reduction of insulin resistance
Cardiovascular disease	• Reduction in cardiovascular deaths • Reduction in myocardial infarction and stroke • Reduction in systolic blood pressure
Liver disease	• 85% NAFLD and NASH resolution • Reduction in histological markers of steatosis, fibrosis, hepatocyte ballooning and lobular inflammation • Reduction in biochemical markers, including AST, ALT, ALP, GGT
Dyslipidemia	• Reduction in LDL cholesterol, VLDL cholesterol, total cholesterol and triglycerides • Increase in HDL cholesterol
Respiratory disease	• Improved respiratory disturbance index • Improved sleep quality (sleep efficiency and rapid eye movement latency) • Reduced requirement for continuous positive airway pressure
Psychosocial disease	• Improved psychosocial functioning and social interaction • Increased physical activity • Reduced depression • Improved health-related quality of life and health perception
Osteoarthritis and chronic back pain	• Reduction in pain • Increase in function/activities of daily living

*NAFLD*, non-alcoholic fatty liver disease; *NASH*, non-alcoholic steatohepatitis; *AST*, aspartate transaminase; *ALT*, alanine transaminase; *ALP*, alkaline phosphatase; *GGT*, gamma-glutamyltransferase

This narrative review discusses the underlying mechanisms that could explain the impact of bariatric surgery and the relationship between obesity and adipose tissue, T2D, gut microbiota and NAFLD.

## Bariatric surgery procedures

Bariatric procedures can be categorized according to their presumed mechanism of action in promoting weight loss. This may consist of malabsorption, gastric restriction or any combination of these mechanisms (Table 3).

**Table 3:** Bariatric surgery procedures

Weight loss mechanism	Procedure	Advantages	Disadvantages/risks
Malabsorptive	JIB	Good weight loss	High risk of nutritional and vitamins deficiencies, diarrhoea, liver failure
Malabsorptive	BPD	Sustained weight loss	High risk of nutritional and vitamins deficiencies, diarrhoea, anemia, intestinal ulcers Complex procedure
Restrictive	VBG	Good weight loss Easy to perform	Long-term weight regain, stapler leak, outlet obstruction, recanalization of proximal stomach, gastro-oesophageal reflux
Restrictive	AGB	Laparoscopic surgery Adjustable, reversible, less pain, fewer nutritional effects	Less weight loss than RYGB, harder to maintain loss, vomiting Band slippage or erosion, tubing breakage
Restrictive	SG	Laparoscopic surgery, Good weight loss Easy to perform Short hospitalization	Irreversible, stapler leak, gastro-oesophageal reflux
Combined	RYGB	Laparoscopic surgery High percentage of weight loss	Vitamin deficiencies Anastomotic leak Internal hernia
Combined	OAGB	Laparoscopic surgery Simpler than RYGB Good weight loss	Vitamin deficiencies Anastomotic leak Less risk of internal hernia
Combined	DS	Sustained weight loss	High risk of nutritional and vitamins deficiencies, loose, foul smelling stools, anemia, intestinal ulcers High complex procedure
Restrictive	BIB	Endoscopic procedure Good weight loss	Temporary device Long-term weight regain

*JIB*, jejunoileal by-pass; *BPD*, biliopancreatic diversion; *VBG*, vertical banded gastroplasty; *AGB*, adjustable gastric banding; *SG*, sleeve gastrectomy; *RYGB*, Roux-en-Y gastric by-pass; *OAGB*, one anastomosis gastric by-pass; *DS*, duodenal switch; *BIB*, bioenteric intragastric balloon

The aim of restrictive procedures is to decrease the amount of ingested food through a reduction of the gastric volume; while in malabsorptive procedures, a part of the small intestine is removed or by-passed, leading to a reduction in gastrointestinal absorptive surface.

JIB was the first pure malabsorptive procedure, but it was burdened with significant complications, including diarrhoea, protein malnutrition, micronutrient and electrolyte deficiencies, and anal complications [7, 9]. Despite this, intestinal by-pass is seldom performed in superobese patients; thus, Scopinaro proposed an intestinal by-pass procedure called BPD [12]. BPD includes a partial gastrectomy with closure of the duodenum and a long intestinal by-pass with a Roux limb of 250-cm length and a 50-cm common channel. The procedure was then modified in DS where the distal gastrectomy was a sleeve gastrectomy, and the common channel had a length of 100 cm [13]. However, surgical complexities and the risk of long-term complications have limited the popularity of these procedures.

Restrictive procedures reduce the stomach capacity by creating a smaller chamber for food intake. One of the earliest restrictive procedures was VBG, which involved the creation of a vertical pouch of about 50-mL volume with an outlet flow encircled by a fixed band to prevent it from dilatation [14]. The concept of an external gastric band sustains the use of AGB. Initially not adjustable, and then adjustable thanks to a subcutaneous port, AGB became popular by laparoscopic approach [15]. However, an overall modest performance and band complications have reduced the number of this procedure over the years. The most recent restrictive procedure is the SG. This procedure was initially performed as the first step of a two-staged DS in high-risk patients who undergo SG and after about 1 year, intestinal by-pass [16]. However, many patients obtained good results with the SG alone; thus, it was adopted as a stand-alone procedure. In SG, the gastric greater curvature is resected, and thus, the stomach is reduced to a narrow tube. The removal of the greater curvature induces weight loss but also hormonal changes, such as a reduction of serum ghrelin levels, which help promote early satiety and prolonged satiation.

RYGB is the most popular version of gastric by-pass, originally proposed by Mason [17]. The procedure, currently considered the “gold standard” in bariatric surgery, includes a vertical lesser curvature pouch, coupled with a jejuno-jejunostomy, in addition to a gastro-jejunostomy and a common limb of around 150 cm. Thus, it is a combination of restrictive and malabsorptive procedures. Of course, the complete procedure of DS, which includes SG and intestinal by-pass, can also be considered a combined restrictive and malabsorptive procedure. Another combined procedure is OAGB, a gastric by-pass that involves only one anastomosis—an end-to-side anastomosis between the gastric pouch and a jejunum loop 150–250 cm from the Treitz ligament [18]. Patients undergoing OAGB were found to have more nutritional deficiencies compared with those who underwent RYGB [19]. To avoid these problems, some surgeons suggest reducing the length of the biliopancreatic limb to less than 150 cm [20].

In recent years, many endoscopic techniques have been proposed to induce weight loss, especially in superobese high-risk patients [21]. The most popular endoscopic technique is the intragastric balloon insertion that decreases the intraluminal gastric volume to induce early satiety during food intake [8]. The device is temporary and must be removed within 6 months, mostly because weight loss is transient. The intragastric balloon, in fact, is used as a bridge to definitive bariatric surgery. Other endoscopic procedures include endoscopic sleeve gastroplasty involving full-thickness sutures and primary obesity surgery endoluminal (POSE) procedure that creates up to ten gastric plications. Both procedures reduce the gastric cavity by remodelling the stomach [21].

## Bariatric surgery and adipose tissue

Adipose tissue is recognized as an endocrine organ implicated in the physiopathology of obesity and its comorbidities [22, 23]. As the organ is a self-contained group of tissues that perform a specific function, in the adipose organ, we can distinguish two different adipose tissues, the white adipose tissue (WAT) storing energy and the brown adipose tissue (BAT) using energy for thermogenesis [24]. The WAT can be divided into two broad categories, visceral adipose tissue (VAT) located in the peritoneal cavity and subcutaneous adipose tissue (SAT) located under the skin. The WAT-BAT cooperation consists of the reciprocal ability of conversion in relation to physiologic requirement of the body [25].

The endocrine function of the adipose tissue is carried out by the secretion of hundreds of different signalling proteins called adipokines into the circulation [26]. These include leptine that suppresses appetite when lipid storage is high and stimulates pro-inflammatory immune response [27] and adiponectine that acts on other organs such as the liver and muscle, and is highly correlated with metabolic derangements of obesity and T2D [28].

Although the adipose organ of animals and humans with obesity is increased at both subcutaneous and visceral sites, VAT alone is responsible for the onset of obesity-associated metabolic disorders [29–31]. These disorders result from adipose tissue dysfunction and inflammation. In obese mice and humans, inflammatory cells infiltrate adipose tissue producing inflammatory mediators that may explain the correlation between visceral fat and cardiovascular and metabolic complications, such as insulin resistance and T2D [3, 32–34]. Macrophages are the main inflammatory cells found in inflamed adipose tissue. Hypertrophic adipocytes die and remnants of dead adipocytes are surrounded by active MAC2 immunoreactive macrophages reabsorbing the large debris. These form a characteristic histopathology feature denominated crown-like structure (CLS) [33]. Furthermore, VAT is composed by more fragile adipocytes compared to those of SAT; in obese subjects, these adipocytes die with a smaller critical death size inducing major inflammation [34].

Why visceral fat behaves differently from SAT in animals and humans with obesity is a concept that is important to understand. The size of adipocytes in VAT is smaller than that of subcutaneous fat. The reason for this difference is not known, but a hypothesis can be proposed. Since a large proportion of VAT in young people is composed by BAT and age is an important factor inducing BAT to WAT conversion, a daring hypothesis for the different size could be that subcutaneous fat originates from WAT adipocyte precursors, while visceral fat originates from conversion of BAT. This theory was recently demonstrated using a mouse-model lacking ATGL (adipose-triglycerides lipase) [3]. In these mice, adipocytes cannot use stored lipids for thermogenesis and BAT is converted into a WAT-like tissue. This WAT-like tissue derived from BAT conversion is more prone to death as shown by the number of CLS in the WAT-like tissue compared with regular WAT with the same size of adipocytes. WAT, producing adipokines, growth factors, enzymes and active immune cells such as macrophages and T cells, takes part in the progression of inflammation in obesity [35]. These data offer an explanation to the higher level of inflammation in VAT and as the visceral obesity is the clinical condition more frequently associated to T2D in obese patients [3].

Studies of depot-specific fat mass show how bariatric surgery induces both VAT and SAT reduction with a metabolically beneficial redistribution among different anatomic depots [36–39]. Through reduction of WAT, bariatric surgery reverses the balance between pro-inflammatory and anti-inflammatory mediators [40, 41]. Recent studies show that post-surgical circulating levels of adiponectin and leptin are significantly increased and decreased respectively [41, 42]. Similarly, serum inflammatory mediators IL-6 and TNF are downregulated [36, 43]. Adiponectin reduces fat storage and inflammation, increases fibrinolysis and additionally activates 5′-AMP-activated protein kinase (AMPK) after surgery. Indeed, subcutaneous adipose tissue levels of AMPK increase after metabolic surgery [44], and AMPK has been associated with improvements in inflammation, oxidative stress, mitochondrial biogenesis and insulin resistance in several tissues [45]. However, it is unclear whether AMPK reduces oxidative stress or whether the reduction of oxidative stress suppresses AMPK [44].

Preclinical and clinical studies suggest that bariatric surgery induces changes in BAT by enhancing thermogenesis [46]. Increasing BAT size has been observed after RYGB [47, 48]. This effect could be due to an increase of glucagon-like peptide 1 (GLP-1) in RYGP, which improves thermogenesis and increases BAT size [49, 50]. On the other hand, no significant changes in BAT size were observed in patients submitted to SG [51]. However, SG may enhance BAT thermogenesis contributing to improve glycaemic control [47, 52]. How BAT activation after surgery modulates the energy balance and remission of T2D is unknown. One possible mechanism may act through bile acids that promote BAT thermogenesis via interaction with the thyroid system and GLP-1 receptor signalling [53, 54].

## Bariatric surgery and T2D

T2D is the most frequent form of diabetes accounting for about 90% of all diagnosis of diabetes. It represents one of the most important pathologies in Western countries, with 592 million T2D patients expected by 2035 [55, 56]. T2D is the fifth leading cause of death [57]. Thus, all efforts to prevent or treat this disease must be encouraged, and all aspects of related scientific research on causes and physiopathology should be strongly sustained by governments worldwide.

T2D is associated with progressive loss of pancreatic beta-cell function and mass [58]. As previously reported, obese fat in mice and humans is infiltrated by macrophages [32, 59]. Macrophages cause a chronic low-grade inflammation producing mainly TNFa and IL6. The mechanism by which tissue inflammation influences insulin sensitivity is unclear, but these molecules have been proven to interfere with the insulin receptor causing insulin resistance [60–63]. The gradual transition of insulin resistance to T2D is linked with a change in pancreatic islet composition. During compensated insulin resistance, pancreatic islets are conspicuous by their hyperplasia, resulting from increased cell number and size [58]. But as compensation fails, islet mass gradually decreases, and beta-cells become depleted of their characteristic insulin secretory granules, ending with a functional exhaustion which coincides with the onset of T2D [58, 63, 64]. Recently, it has been demonstrated that obese mice have an increased noradrenergic innervation of Langerhans islets, with data supporting nerve-epithelial contacts with beta-cells [58, 65]. A recent paper confirmed these data in humans [66]. Thus, considering the well-known inhibitory activity of noradrenaline on insulin secretion, the hypothesis is that the lack of insulin secretion inducing T2D after a period of hyper-production is not due to beta-cell exhaustion, but to beta-cell inhibition by increased noradrenergic innervation. For unknown reasons, bariatric surgery could induce a de-innervation process in the Langerhans islets restoring the insulin secretory activity of beta-cells. Data supporting a direct innervation of pancreatic islets by neurons located in the intestinal wall are in line with the idea that surgical removal or intestinal by-pass could influence pancreatic islet innervation [67].

In the last decade, a considerable amount of high-quality evidence supported that bariatric surgery has an effective role in the treatment of T2D, with an improvement of glucometabolic profiles and a complete remission of diabetes [68]. The effects of metabolic surgery are stable over time, with a substantially greater effect at five years compared with medical treatment [69]. Some factors contribute to the post-operative diabetes response after surgery. Patients with a BMI > 30 kg/m^2^ and those with a BMI < 30 kg/m^2^ have distinct remission predicting factors. Low HbA1c is a predictor of remission in low–high-BMI patients while the length of time in which the patients are affected by diabetes is a predictor in high-low-BMI patients [70].

**Fig. 1:**
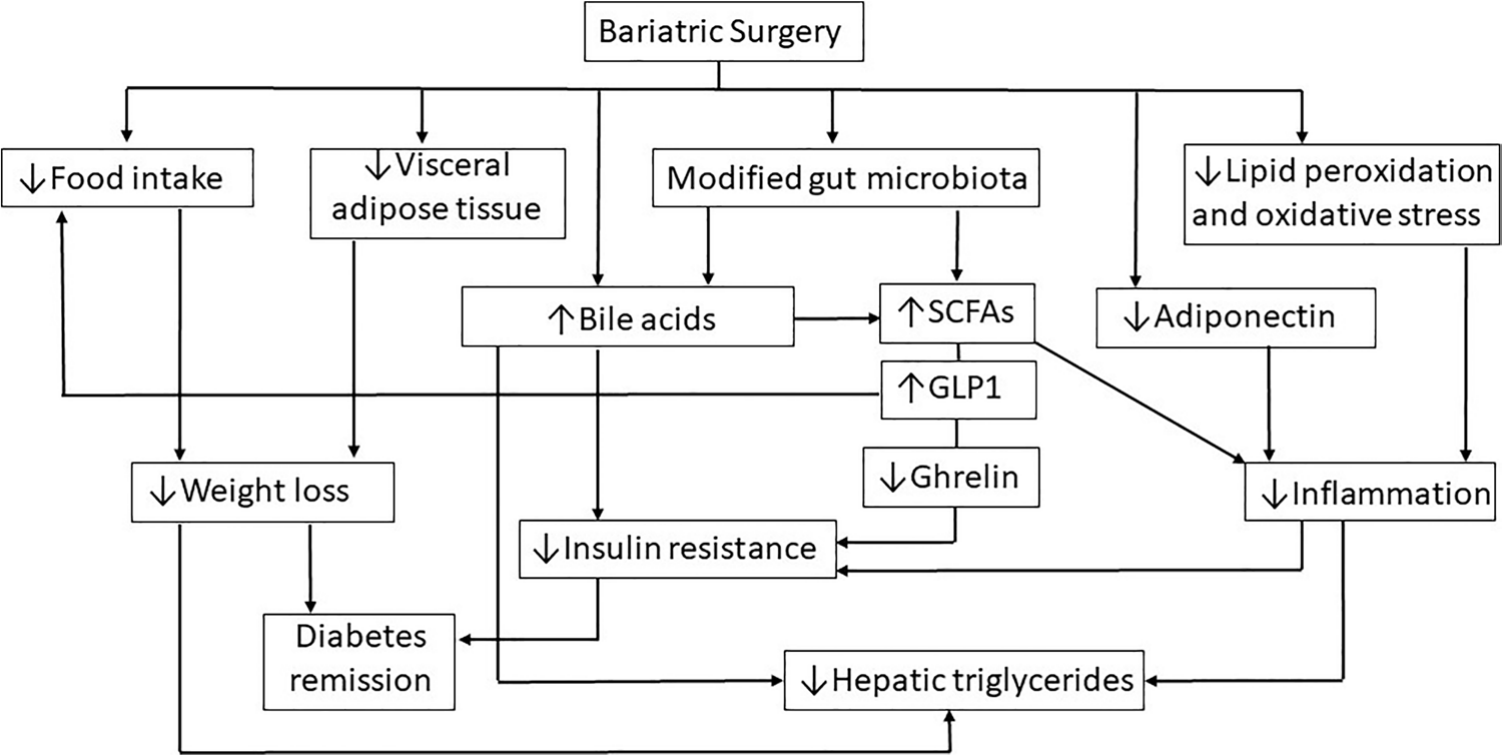
The cross-linking between bariatric surgery and adipose tissue, glucose metabolism, gut microbiota and NAFLD. Restrictive and/or malabsorptive surgeries such as SG and RYGB have important effects on lipid and glucose metabolism by direct action or through modification of gut microbiota. SCFAs, short-chain fatty acids; GLP1, glucagon-like peptide 1

In fact, in animal models of obesity and in humans undergoing different types of bariatric surgery, T2D improves within days to weeks, whereas weight loss occurs much more slowly [68]. The weight-independent mechanisms involved are not completely understood, but they include the incretin effect of GLP1, alterations in bile acids and changes in gut microbiota composition [5]. GLP1 increases dramatically after bariatric surgery, independently of both calorie reduction and weight loss [71]. Therefore, reduction of food intake (anorexic effect of surgery), reduction of insulin resistance (long-term weight reduction after surgery) and increase of insulin secretion (which is weight loss independent) improves diabetes. Bile acids probably interact with the gut microbiota in the duodenum and proximal jejunum. The gut microbiota has an interdependent relationship with bile acids, whereby bile acids affect the microbiota composition by altering bacterial membrane integrity, and the gut microbiota can alter bile acid synthesis and function, including bile acid deconjugation, dihydroxylation, oxidation and epimerization [72, 73]. The effects and interactions of these systems are illustrated in Fig. 1.

## Bariatric surgery and gut microbiota

The human gut, mainly the colon, holds the greatest numbers of microbiota in the organism. Humans and microorganisms have long benefited from this symbiotic relationship, yet our understanding of the extent and meaning of this co-existence has been limited due to the lack of reliable and effective tools to study it. Recent evidence has suggested a role for alterations in the gut microbiota in promoting or aggravating different diseases such as obesity [74].

Components of gut microbiota are now considered to play a significant role in several fields, such as the regulation of intestinal function, metabolism, behaviour and immunity [75].

The adult gut microbiota is dominated by two phyla, *Firmicutes* and *Bacteroidetes*, which constitute about 90% of all the bacterial species in the gut [74]. Many studies have shown a relative decrease of *Bacteroidetes* with a relative increase in *Firmicutes* in the obese microbiota, but the findings are still weak [76–79]. A difficult question is whether changes in the intestinal microbiota precede the development of obesity or reflect the obese phenotype. Of course, diet, microbiota and immunity participate in the development of obesity [80–83].

Several theories discuss the hypothesis that gut microbiota can induce obesity. One hypothesis is that the gut microbiota of individuals with obesity is capable of fermenting dietary carbohydrates that increases the rate of short-chain fatty acids (SCFAs), providing extra energy, which are then stored as lipids or glucose [77, 78, 84]. In fact, *Firmicutes*, which are major producers of the SCFAs, are increased in the obese faecal microbiota [76]. This theory is supported by transplanting obese faecal microbiota in germ-free mice and finding a higher level of *Firmicutes* in faecal samples and an increase in body fat [76]. Carbohydrate and lipid metabolism are highly influenced by microbiota that increase the bioavailability of monosaccharides, and the subsequent induction of de novo hepatic lipogenesis. The microbiota of genetically obese mice is rich in enzymes involved in the fermentation of dietary fibre; the products of dietary fibre fermentation include SCFAs such as acetate, propionate and butyrate [74], which generally improve glucose and energy homeostasis [85]. Preclinical and human studies show that obese individuals present higher faecal concentrations of SCFAs than lean controls [75, 86]. However, the role of SCFAs is controversial because obesity-inhibiting properties have been described [87]. SCFAs suppress the inflammatory immune response in the gut [88, 89] and they are involved in the release of GLP-1 and leptine, which may downregulate appetite and thus reduce caloric intake [90].

Weight loss interventions, as bariatric surgery, induce a decrease in faecal SCFAs, mainly due to low-carbohydrate diets [87]. This reduction could be an indication of reduced efficiency with which energy is harvested from dietary SCFAs during weight loss among overweight or obese individuals [75, 87]. Given the benefits of SCFA on colon cancer risk [91], studies are needed to clarify if the decrease of SCFA is a potential adverse effect of weight loss.

Another theory is that a key process in the biological physiology of obesity includes systemic inflammatory changes.

The systemic increased levels of adiponectin and inflammatory cytokines, such as tumour necrosis factor-alpha (TNF-α) and interleukin-6 (IL-6), has also been associated with hypertrophy of adipose tissue and with augmented risk of metabolic disorders such as cardiovascular diseases, fatty liver disease and T2D [74]. Despite these various association studies, the causal pathways between obesity, inflammation and metabolic disease remain incompletely understood. The presence of low-grade systemic inflammation associated with obesity usually involves a complex network of signals interconnecting several organs [92, 93]. Understanding the mechanisms that regulate gut microbiota homeostasis and dysbiosis will lead to a better comprehension of the inflammation-related pathophysiology of obesity and consequently could provide an avenue for interventions aimed at modulating gut microbiota in individuals with obesity [94, 95].

The ingestion of high fat diets could be a facilitating factor in the disruptions of gut microbiota homeostasis in obesity [74, 95]. These diet-induced changes in the microbiota physiology can cause low-grade systemic inflammation in obesity and may even precede or predispose to obesity [94–98]. Changes in the composition of gut microbiota increase intestinal mucosal inflammation with changes in gut permeability. Together, these processes can result in increased metabolic endotoxemia and in an increase of components such as plasma lipopolysaccharides (LPS) within the circulating system [99]. The gut microbiota-related inflammatory changes have been linked to activation of toll-like receptor 4 (TLR4) signalling and to a resulting increase in intestinal levels of LPS [100]. Studies have also shown that increased levels of LPS, together with TLR4, are risk factors for obesity, insulin resistance and cardiovascular diseases [101–103].

Significant changes in gut microbiota have been noted after bariatric surgery, specifically with increases in *Bacteroidetes*, *Fusobacteria*, *Verrucomicrobia* and *Proteobacteria* and a reduction of *Firmicutes*, *Clostridiales*, *Clostridiaceae*, *Blautia* and *Dorea* [55, 104, 105]. An increase in *Bacteroidetes* species has been correlated with a reduction in body fat mass and leptin, while the *Firmicutes* responsible for dietary carbohydrate fermentation and energy harvesting are decreased [106, 107].

Most of the changes in microbial composition occurred within 3 months and those changes were maintained up to a year [108]. This points out how remodelling of the microbial community occurred mainly within the first 3 months after surgery [108].

Gut microbiota modifications are different between bariatric procedures [109]. *Bacteroides vulgatus*, a bacteria increased in patients with obesity and positively correlated with glycaemic status, is reduced significantly after SG, whereas it is not significantly affected by either post-AGB or post-RYGB [110]. Furthermore, SG also increases *Faecalibacterium prausnitzii*, another bacterium decreased in obese subjects with T2D that increases post-RYGB [111]. These data allow hypothesizing that the change in these bacteria could be involved in the glucose improvement observed after SG; however, this is still unclear. In another study, comparing SG and RYGB in a small sample size, Murphy et al. observed that although SG was associated with functional changes in gut microbiota, these were fewer than those observed post-RYGB43. Furthermore, in another study comparing SG and RYGB, both procedures induced similar clinical improvement, but gut microbiota modifications involved distinct pathways according to the surgical technique [112].

Post-RYGB patient gut microbiota reduced body weight gain when transferred into germ-free mice. These effects appear to be part of the weight loss-independent mechanisms of RYGB, as the germ-free mice colonized with the microbiota from post-RYGB patients gained 43% less body fat than mice colonized with the microbiota from weight-matched patients who did not have surgery [113].

RYGB produces significant metabolic changes, including decrease in plasma bile acid content and increases in various amines production, which reflect changes in the microbial metabolism of precursors like choline [114].

Bariatric surgery affects bile acids (BAs) metabolism [110]. BAs influence glucose metabolism by increasing insulin sensitivity and reducing gluconeogenesis [115] through increased secretion of GLP-1 and activation of TGR5, improving the energy balance [116]. Moreover, BAs play a role in the gut microbiota composition and in the weight loss after bariatric surgery [117]. A recent study conducted by Ilhan et al. focused attention on the gut microbiota of obese patients who had underwent RYGB. Surgery causes a reduction in faecal BA concentration, which relates to changes in microbiota composition, and the gut microbiota itself is involved in the modulation of BAs metabolism [118]. In fact, the anatomical changes made in RYGB increase the amount of BAs reaching the lower intestine, thus allowing conjugated BAs to be actively reabsorbed in the terminal ileum and primary BAs to enter the colon and be transformed into secondary BAs by the gut microbiota [111]. These changes are linked with fatty liver disease. In NAFLD patients, the serum primary/secondary BAs ratio is significantly higher compared to controls and correlates with the severity of NAFLD [119]. Bariatric surgery produces a significant repopulation of the gut microbiota and a reversal of the circulating primary/secondary BAs ratio, thus inducing metabolic improvements with positive effects on NAFLD and metabolic syndrome [119].

## Bariatric surgery and NAFLD

NAFLD is defined as more than 5% fat accumulation in hepatocytes [120]. In adult population, NAFLD has a high prevalence, especially in people with obesity (65.7%) and T2D (74%) [121–123]. The association between NAFLD and T2D can be explained by insulin resistance, dyslipidaemia and the accumulation of liver triglycerides in NAFLD and beta-cell defect in T2D [124]. NAFLD can progress from simple hepatic steatosis to steatohepatitis (NASH), which is characterized by inflammation and hepatocyte degeneration. A small proportion of NASH will further progress to liver fibrosis/cirrhosis and hepatocellular carcinoma. Obesity is a dominant risk factor for development of NAFLD and NASH, with 4.5-fold increased risk of hepatocellular carcinoma [125].

Secretion of adropin, an insulin sensitizing factor, and of sex hormone-binding globulin (SHBG) can be observed in the liver affected by NAFLD [41]. Lower levels of SHBG could be involved in the development of NAFLD and T2D, but data are still unclear [41].

Many studies have shown that dysregulation of the gut microbiota can be involved in the pathogenesis of NAFLD [126–128]. Changes in the abundance and diversity of the gut microbiota have been linked to the progression of NAFLD; each stage of NAFLD has a special gut microbiota signature [129].

In NAFLD, *Bacteroidetes* are reported to be decreased, while levels of *Firmicutes* and *Proteobacteria* are increased, especially in patients with obesity [126, 129, 130]. Changes in microbiota in NASH are reported to overlap with steatosis, with differences identified especially in patients diagnosed with NASH with fibrosis. For example, *Eubacterium rectale* is increased in moderately severe NAFLD, but decreased in NASH with fibrosis [129]. The higher the degree of fibrosis, the higher the abundance of *Proteobacteria*, and this suggests the role of these bacteria in the process of liver fibrosis, although the exact mechanism is still unknown [131].

The expression of genes involved in LPS synthesis in gut microbiota is increased in NASH compared with steatosis, while increased flagellar biosynthesis gene expression in NASH indicates fibrosis. Furthermore, bacterial translocation due to increased gut permeability and increased blood levels of LPS have been associated with NAFLD [132, 133].

The intestinal microbiota has the ability which seems to play a role in the pathogenesis of NAFLD, through different pathways of bacterial metabolites such as bile acids, SCFAs, amino acids, choline and ethanol [129].

Weight loss is currently the mainstay of NAFLD treatment. A 3 to 5% weight loss has been shown to reduce steatosis, and a greater weight loss of up to 10% might be necessary to improve hepatic necro-inflammation [134]. However, most NAFLD patients are not able to achieve such weight loss by diet restriction, but bariatric surgery can produce up to 85% resolution of NAFLD and NASH, with an improvement of both histological and biochemical markers [135]. Oxidative stress and lipid peroxidation in patients with NAFLD also improve after metabolic surgery, reducing DNA damage and the inflammatory cascade from hepatocellular injury to fibrosis and cirrhosis [135].

Analysing the effect of specific bariatric procedures, SG determines improvements in aspartate aminotransferase, alanine aminotransferase, triglycerides and high-density lipoprotein serum levels and the NAFLD resolution assessed with ultrasound imaging and histological amelioration [136–139]. RYGB leads to reduction of steatosis, lobular inflammation, ballooning degeneration and centrilobular/perisinusoidal fibrosis [140–143]. Several studies suggest that RYGB is more effective compared with SG and LAGB in terms of benefits on NAFLD, NASH and fibrosis, whereas, in other studies, SG shows a better improvement than RYGB in serum levels of hepatic enzymes [136, 144, 145].

As mentioned above, bariatric surgery has additional effects beyond weight loss, which contribute to amelioration of NAFLD. Post-surgical changes in the gut microbiota and bile acid circulation, as well as a decrease in portal influx of free fatty acids, may also be beneficial for metabolic syndrome and NAFLD, as suggested by recent studies [115, 146–148].

## Future perspective

A substantial number of metabolic modifications occurs after bariatric surgery. The mechanism involved might be either weight dependent or weight independent. The cross-linking of these mechanisms is key in the long-term effects after surgery. Inflammation in visceral fat is strictly connected with insulin resistance and T2D, but it is also related with gut microbiota. However, human studies on changes in the gut microbiota are still relatively unpowered, are not always conclusive and vary across different populations. For these reasons, further research is needed to investigate how microbiota modifications are related to glucose and lipid metabolism after bariatric surgery.

## Data Availability

All data generated or analysed during this study are included in this published article and the reference list.

## References

[1] NCD Risk Factor Collaboration (NCD-RisC) (2016) Trends in adult body-mass index in 200 countries from 1975 to 2014: a pooled analysis of 1698 population-based measurement studies with 192 million participants. Lancet 387:1377–1396 10.1016/S0140-6736(16)30054-X PMC7615134 27115820

[2] Klöting N, Fasshauer M, Dietrich A, Kovacs P, Schön MR, Kern M, Stumvoll M, Blüher M (2010). Insulin-sensitive obesity. Am J Physiol Endocrinol Metab.

[3] Kotzbeck P, Giordano A, Mondini E, Murano I, Severi I, Venema W, Cecchini MP, Kershaw EE, Barbatelli G, Haemmerle G, Zechner R, Cinti S (2018). Brown adipose tissue whitening leads to brown adipocyte death and adipose tissue inflammation. J Lipid Res.

[4] Schauer PR, Bhatt DL, Kirwan JP, Wolski K, Brethauer SA, Navaneethan SD, Aminian A, Pothier CE, Kim ES, Nissen SE, Kashyap SR (2014). STAMPEDE Investigators. Bariatric surgery versus intensive medical therapy for diabetes-3-year outcomes. N Engl J Med.

[5] Sinclair P, Brennan DJ, le Roux CW (2018). Gut adaptation after metabolic surgery and its influences on the brain, liver and cancer. Nat Rev Gastroenterol Hepatol.

[6] Buchwald H (2014). The evolution of metabolic/bariatric surgery. Obes Surg.

[7] Wiggins T, Majid MS, Agrawal S (2020). From the Knife to the Endoscope-a History of Bariatric Surgery. Curr Obes Rep.

[8] Angrisani L, Santonicola A, Iovino P, Ramos A, Shikora S, Kow L (2021). Bariatric surgery survey 2018: similarities and disparities among the 5 IFSO chapters. Obes Surg.

[9] Phillips BT, Shikora SA (2018). The history of metabolic and bariatric surgery: development of standards for patient safety and efficacy. Metab.

[10] Wolfe BM, Kvach E, Eckel RH (2016). Treatment of obesity: weight loss and bariatric surgery. Circ Res.

[11] Hacken B, Rogers A, Chinchilli V, Silvis M, Mosher T, Black K (2019). Improvement in knee osteoarthritis pain and function following bariatric surgery: 5-year follow-up. Surg Obes Relat Dis.

[12] Scopinaro N, Gianetta E, Civalleri D, Bonalumi U, Bachi V (1979). Bilio-pancreatic bypass for obesity: II. Initial experience in man. Br J Surg.

[13] Hess DS, Hess DW (1998). Biliopancreatic diversion with a duodenal switch. Obes Surg.

[14] Printen KJ, Mason EE (1973). Gastric surgery for relief of morbid obesity. Arch Surg.

[15] O’Brien PE, MacDonald L, Anderson M, Brennan L, Brown WA (2013). Long-term outcomes after bariatric surgery: fifteen-year follow-up of adjustable gastric banding and a systematic review of the bariatric surgical literature. Ann Surg.

[16] Regan JP, Inabnet WB, Gagner M, Pomp A (2003). Early experience with two-stage laparoscopic Roux-en-Y gastric bypass as an alternative in the super-super obese patient. Obes Surg.

[17] Mason EE, Ito C (1967). Gastric bypass in obesity. Surg Clin North Am.

[18] Rutledge R, Walsh TR (2005). Continued excellent results with the mini-gastric bypass: six-year study in 2,410 patients. Obes Surg.

[19] Robert M, Espalieu P, Pelascini E, Caiazzo R, Sterkers A, Khamphommala L, Poghosyan T, Chevallier JM, Malherbe V, Chouillard E, Reche F, Torcivia A, Maucort-Boulch D, Bin-Dorel S, Langlois-Jacques C, Delaunay D, Pattou F, Disse E (2019). Efficacy and safety of one anastomosis gastric bypass versus Roux-en-Y gastric bypass for obesity (YOMEGA): a multicentre, randomised, open-label, non-inferiority trial. Lancet.

[20] Hussain A, Van den Bossche M, Kerrigan DD, Alhamdani A, Parmar C, Javed S, Harper C, Darrien J, Singhal R, Yeluri S, Vasas P, Balchandra S, El-Hasani S (2019). Retrospective cohort study of 925 OAGB procedures. The UK MGB/OAGB collaborative group. Int J Surg.

[21] Saunders KH, Igel LI, Saumoy M, Sharaiha RZ, Aronne LJ (2018). Devices and endoscopic bariatric therapies for obesity. Curr Obes Rep.

[22] Cinti S (2018). Adipose organ development and remodeling. Compr Physiol.

[23] Vitali A, Murano I, Zingaretti MC, Frontini A, Ricquier D, Cinti S (2012). The adipose organ of obesity-prone C57BL/6J mice is composed of mixed white and brown adipocytes. J Lipid Res.

[24] Ronkainen J, Mondini E, Cinti F, Cinti S, Sebért S, Savolainen MJ, Salonurmi T (2016). Fto-deficiency affects the gene and MicroRNA expression involved in brown adipogenesis and browning of white adipose tissue in mice. Int J Mol Sci.

[25] Rodriguez A, Catalan V, Gomez-Ambrosi J, Fruhbeck G (2007). Visceral and subcutaneous adiposity: are both potential therapeutic targets for tackling the metabolic syndrome?. Curr Pharm Des.

[26] Lehr S, Hartwig S, Sell H (2012). Adipokines: a treasure trove for the discovery of biomarkers for metabolic disorders. Proteomics Clin Appl.

[27] La Cava A, Alviggi C, Matarese G (2004). Unraveling the multiple roles of leptin in inflammation and autoimmunity. J Mol Med.

[28] Mohan V, Deepa R, Pradeepa R (2005). Association of low adiponectin levels with the metabolic syndrome—the Chennai Urban Rural Epidemiology Study (CURES-4). Metab.

[29] Thörne A, Lönnqvist F, Apelman J, Hellers G, Arner P (2002). A pilot study of long-term effects of a novel obesity treatment: omentectomy in connection with adjustable gastric banding. Int J Obes Relat Metab Disord.

[30] Wajchenberg BL (2000). Subcutaneous and visceral adipose tissue: their relation to the metabolic syndrome. Endocr Rev.

[31] Xu H, Barnes GT, Yang Q, Tan G, Yang D, Chou CJ, Sole J, Nichols A, Ross JS, Tartaglia LA, Chen H (2003). Chronic inflammation in fat plays a crucial role in the development of obesity-related insulin resistance. J Clin Invest.

[32] Cinti S, Mitchell G, Barbatelli G, Murano I, Ceresi E, Faloia E, Wang S, Fortier M, Greenberg AS, Obin MS (2005). Adipocyte death defines macrophage localization and function in adipose tissue of obese mice and humans. J Lipid Res.

[33] Murano I, Barbatelli G, Parisani V, Latini C, Muzzonigro G, Castellucci M, Cinti S (2008). Dead adipocytes, detected as crown-like structures, are prevalent in visceral fat depots of genetically obese mice. J Lipid Res.

[34] Zimmet P, Shaw J (2017). Diabetes: rising incidence of diabetes mellitus in youth in the USA. Nat Rev Endocrinol.

[35] Exley MA, Hand L, O’Shea D, Lynch L (2014). Interplay between the immune system and adipose tissue in obesity. J Endocrinol.

[36] Frikke-Schmidt H, O’Rourke RW, Lumeng CN, Sandoval DA, Seeley RJ (2016). Does bariatric surgery improve adipose tissue function?. Obes Rev.

[37] Galanakis CG, Daskalakis M, Manios A, Xyda A, Karantanas AH, Melissas J (2014). Computed tomography-based assessment of abdominal adiposity changes and their impact on metabolic alterations following bariatric surgery. World J Surg.

[38] Toro-Ramos T, Goodpaster BH, Janumala I (2015). Continued loss in visceral and intermuscular adipose tissue in weight-stable women following bariatric surgery. Obes.

[39] Keidar A, Appelbaum L, Schweiger C (2014). Baseline abdominal lipid partitioning is associated with the metabolic response to bariatric surgery. Obes Surg.

[40] Labrecque J, Laforest S, Michaud A, Biertho L, Tchernof A (2017). Impact of bariatric surgery on white adipose tissue inflammation. Can J Diabetes.

[41] Faramia J, Ostinelli G, Drolet-Labelle V, Picard F, Tchernof A (2020). Metabolic adaptations after bariatric surgery: adipokines, myokines and hepatokines. Curr Opin Pharmacol.

[42] Khosravi-Largani M, Nojomi M, Aghili R (2019). Evaluation of all types of metabolic bariatric surgery and its consequences: a systematic review and meta-analysis. OBES SURG.

[43] Moschen AR, Molnar C, Geiger S, Graziadei I, Ebenbichler CF, Weiss H, Kaser S, Kaser A, Tilg H (2010). Anti-inflammatory effects of excessive weight loss: potent suppression of adipose interleukin 6 and tumour necrosis factor alpha expression. Gut.

[44] Xu XJ, Apovian C, Hess D, Carmine B, Saha A, Ruderman N (2015). Improved insulin sensitivity 3 months after RYGB surgery is associated with increased subcutaneous adipose tissue AMPK activity and decreased oxidative stress. Diabetes.

[45] Salminen A, Hyttinen JM, Kaarniranta K (2011). AMP-activated protein kinase inhibits NF-κB signaling and inflammation: impact on healthspan and lifespan. J Mol Med.

[46] Hankir MK, Seyfried F (2020). Do bariatric surgeries enhance brown/beige adipose tissue thermogenesis?. Front Endocrinol.

[47] Dadson P, Hannukainen JC, Din MU, Lahesmaa M, Kalliokoski KK, Iozzo P (2018). Brown adipose tissue lipid metabolism in morbid obesity: effect of bariatric surgery-induced weight loss. Diabetes Obes Metab.

[48] Vijgen GH, Bouvy ND, Teule GJ (2012). Increase in brown adipose tissue activity after weight loss in morbidly obese subjects. J Clin Endocrinol Metab.

[49] Krieger JP, Santos da Conceicao EP, Sanchez-Watts G (2018). Glucagon-like peptide-1 regulates brown adipose tissue thermogenesis via the gut-brain axis in rats. Am J Physiol Regul Integr Comp Physiol..

[50] Chen Y, Yang J, Nie X (2018). Effects of bariatric surgery on change of Brown adipocyte tissue and energy metabolism in obese mice. Obes Surg.

[51] Hui SCN, Wong SKH, Ai Q (2019). Observed changes in brown, white, hepatic and pancreatic fat after bariatric surgery: evaluation with MRI. Eur Radiol.

[52] Piquer-Garcia I, Cereijo R, Corral-Perez J, Pellitero S, Martinez E, Taxeras SD (2020). Use of infrared thermography to estimate brown fat activation after a cooling protocol in patients with severe obesity that underwent bariatricsurgery. Obes Surg.

[53] Watanabe M, Houten SM, Mataki C, Christoffolete MA, Kim BW, Sato H, Messaddeq N, Harney JW, Ezaki O, Kodama T, Schoonjans K, Bianco AC, Auwerx J (2006). Bile acids induce energy expenditure by promoting intracellular thyroid hormone activation. Nature.

[54] Villarroya F, Vidal-Puig A (2013). Beyond the sympathetic tone: the new brown fat activators. Cell Metab.

[55] Boles A, Kandimalla R, Reddy PH (2017). Dynamics of diabetes and obesity: Epidemiological perspective. Biochim Biophys Acta Mol Basis Dis.

[56] Shaw JE, Sicree RA, Zimmet PZ (2010). Global estimates of the prevalence of diabetes for 2010 and 2030. Diabetes Res Clin Pract.

[57] Giannulis I, Mondini E, Cinti F, Frontini A, Murano I, Barazzoni R, Barbatelli G, Accili D, Cinti S (2014). Increased density of inhibitory noradrenergic parenchymal nerve fibers in hypertrophic islets of Langerhans of obese mice. Nutr Metab Cardiovasc Dis.

[58] Weisberg SP, McCann D, Desai M, Rosenbaum M, Leibel RL, Ferrante AW (2003). Obesity is associated with macrophage accumulation in adipose tissue. J Clin Invest.

[59] Hotamisligil GS (2017). Inflammation, metaflammation and immunometabolic disorders. Nature.

[60] Ying W, Riopel M, Bandyopadhyay G, Dong Y, Birmingham A, Seo JB, Ofrecio JM, Wollam J, Hernandez-Carretero A, Fu W, Li P, Olefsky JM (2017). Adipose tissue macrophage-derived exosomal miRNAs can modulate in vivo and in vitro insulin sensitivity. Cell.

[61] Nigi L, Grieco GE, Ventriglia G, Brusco N, Mancarella F, Formichi C, Dotta F, Sebastiani G (2018). MicroRNAs as regulators of insulin signaling: research updates and potential therapeutic perspectives in type 2 diabetes. Int J Mol Sci.

[62] Ferrannini E (1997). Insulin resistance is central to the burden of diabetes. Diabetes Metab Rev.

[63] Kitamura Y, Accili D (2004). New insights into the integrated physiology of insulin action. Rev Endocr Metab Disord.

[64] Xuan S, Szabolcs M, Cinti F, Perincheri S, Accili D, Efstratiadis A (2010). Genetic analysis of type-1 insulin-like growth factor receptor signaling through insulin receptor substrate-1 and -2 in pancreatic beta cells. J Biol Chem.

[65] Cinti F, Bouchi R, Kim-Muller JY, Ohmura Y, Sandoval PR, Masini M, Marselli L, Suleiman M, Ratner LE, Marchetti P, Accili D (2016). Evidence of β-cell dedifferentiation in human type 2 diabetes. J Clin Endocrinol Metab.

[66] Lin EE, Scott-Solomon E, Kuruvilla R (2021). Peripheral innervation in the regulation of glucose homeostasis. Trends Neurosci.

[67] Pareek M, Schauer PR, Kaplan LM, Leiter LA, Rubino F, Bhatt DL (2018). Metabolic surgery: weight loss, diabetes, and beyond. J Am Coll Cardiol.

[68] Bose M, Teixeira J, Olivan B, Bawa B, Arias S, Machineni S, Pi-Sunyer FX, Scherer PE, Laferrère B (2010). Weight loss and incretin responsiveness improve glucose control independently after gastric bypass surgery. J Diabetes.

[69] Sheng B, Truong K, Spitler H, Zhang L, Tong X, Chen L (2017). The long-term effects of bariatric surgery on type 2 diabetes remission, microvascular and macrovascular complications, and mortality: a systematic review and meta-analysis. Obes Surg.

[70] Liang H, Cao Q, Liu H, Guan W, Wong C, Tong D (2018). The predictive factors for diabetic remission in Chinese patients with BMI > 30 kg/m^2^ and BMI < 30 kg/m^2^ are different. Obes Surg.

[71] Park MY, Kim SJ, Ko EK, Ahn SH, Seo H, Sung MK (2016). Gut microbiota- associated bile acid deconjugation accelerates hepatic steatosis in ob/ob mice. J Appl Microbiol.

[72] Zheng X, Huang F, Zhao A, Lei S, Zhang Y, Xie G, Chen T, Qu C, Rajani C, Dong B, Li D, Jia W (2017). Bile acid is a significant host factor shaping the gut microbiome of diet- induced obese mice. BMC Biol.

[73] Sanmiguel C, Gupta A, Mayer EA (2015). Gut microbiome and obesity: a plausible explanation for obesity Curr Obes Rep.

[74] Portune KJ, Benìtez-Pàez A, Del Pulgar EM, Cerrudo V, Sanz Y (2017) Gut microbiota, diet, and obesity-related disorders-The good, the bad, and the future challenges. Mol Nutr Food Res 61(1):1600252 10.1002/mnfr.201600252 27287778

[75] Turnbaugh PJ, Ley RE, Mahowald MA, Magrini V, Mardis ER, Gordon JI (2006). An obesity-associated gut microbiome with increased capacity for energy harvest. Nature.

[76] Ley RE, Turnbaugh PJ, Klein S, Gordon JI (2006). Microbial ecology: human gut microbes associated with obesity. Nature.

[77] Turnbaugh PJ, Hamady M, Yatsunenko T, Cantarel BL, Duncan A, Ley RE, Sogin ML, Jones WJ, Roe BA, Affourtit JP, Egholm M, Henrissat B, Heath AC, Knight R, Gordon JI (2009). A core gut microbiome in obese and lean twins. Nature.

[78] Turnbaugh PJ, Ridaura VK, Faith JJ, Rey FE, Knight R, Gordon JI (2009). The effect of diet on the human gut microbiome: a metagenomic analysis in humanized gnotobiotic mice. Sci Transl Med.

[79] Blustein J, Attina T, Liu M, Ryan AM, Cox LM, Blaser MJ, Trasande L (2013). Association of caesarean delivery with child adiposity from age 6 weeks to 15 years. Int J Obes.

[80] Bergström A, Skov TH, Bahl MI, Roager HM, Christensen LB, Ejlerskov KT, Mølgaard C, Michaelsen KF, Licht TR (2014). Establishment of intestinal microbiota during early life: a longitudinal, explorative study of a large cohort of Danish infants. Appl Environ Microbiol.

[81] Graff M, Ngwa JS, Workalemahu T, Homuth G, Schipf S, Teumer A, Völzke H, Wallaschofski H, Abecasis GR, Edward L, Francesco C, Sanna S, Scheet P, Schlessinger D, Sidore C, Xiao X, Wang Z, Chanock SJ, Jacobs KB, Hayes RB, Hu F, Van Dam RM; GIANT Consortium, Crout RJ, Marazita ML, Shaffer JR, Atwood LD, Fox CS, Heard-Costa NL, White C, Choh AC, Czerwinski SA, Demerath EW, Dyer TD, Towne B, Amin N, Oostra BA, Van Duijn CM, Zillikens MC, Esko T, Nelis M, Nikopensius T, Metspalu A, Strachan DP, Monda K, Qi L, North KE, Cupples LA, Gordon-Larsen P, Berndt SI (2013). Genome-wide analysis of BMI in adolescents and young adults reveals additional insight into the effects of genetic loci over the life course. Hum Mol Genet.

[82] Ridaura VK, Faith JJ, Rey FE, Cheng J, Duncan AE, Kau AL, Griffin NW, Lombard V, Henrissat B, Bain JR, Muehlbauer MJ, Ilkayeva O, Semenkovich CF, Funai K, Hayashi DK, Lyle BJ, Martini MC, Ursell LK, Clemente JC, Van Treuren W, Walters WA, Knight R, Newgard CB, Heath AC, Gordon JI (2013). Gut microbiota from twins discordant for obesity modulate metabolism in mice. Sci.

[83] Trasande L, Blustein J, Liu M, Corwin E, Cox LM, Blaser MJ (2013). Infant antibiotic exposures and early-life body mass. Int J Obes.

[84] Muscogiuri G, Cantone E, Cassarano S, Tuccinardi D, Barrea L, Savastano S, Colao A (2019). on behalf of the Obesity Programs of nutrition, Education, Research and Assessment (OPERA) group. Gut microbiota: a new path to treat obesity. Int J Obes Suppl.

[85] Samuel BS, Shaito A, Motoike T, Rey FE, Backhed F, Manchester JK, Hammer RE, Williams SC, Crowley J, Yanagisawa M, Gordon JI (2008). Effects of the gut microbiota on host adiposity are modulated by the short-chain fatty-acid binding G protein-coupled receptor, Gpr41. Proc Natl Acad Sci U S A.

[86] Schwiertz A, Taras D, Schäfer K, Beijer S, Bos NA, Donus C, Hardt PD (2010). Microbiota and SCFA in lean and overweight healthy subjects. Obesity.

[87] Sowah SA, Riedl L, Damms-Machado A, Johnson TS, Schübel R, Graf M, Kartal E, Zeller G, Schwingshackl L, Stangl GI, Kaaks R, Kühn T (2019). Effects of weight-loss interventions on short-chain fatty acid concentrations in blood and feces of adults: a systematic review. Adv Nutr.

[88] Maslowski KM, Vieira AT, Ng A, Kranich J, Sierro F, Yu D, Schilter HC, Rolph MS, Mackay F, Artis D, Xavier RJ, Teixeira MM, Mackay CR (2009). Regulation of inflammatory responses by gut microbiota and chemoattractant receptor GPR43. Nature.

[89] Kimura I, Ozawa K, Inoue D, Imamura T, Kimura K, Maeda T, Terasawa K, Kashihara D, Hirano K, Tani T, Takahashi T, Miyauchi S, Shioi G, Inoue H, Tsujimoto G (2013). The gut microbiota suppresses insulin-mediated fat accumulation via the short-chain fatty acid receptor GPR43. Nat Commun.

[90] Chambers ES, Viardot A, Psichas A, Morrison DJ, Murphy KG, ZacVarghese SE, MacDougall K, Preston T, Tedford C, Finlayson GS (2015). Effects of targeted delivery of propionate to the human colon on appetite regulation, body weight maintenance and adiposity in overweight adults. Gut.

[91] Zeng H, Umar S, Rust B, Lazarova D, Bordonaro M (2019). Secondary bile acids and short chain fatty acids in the colon: a focus on colonic microbiome, cell proliferation, inflammation, and cancer. Int J Mol Sci.

[92] Sell H, Eckel J (2010). Adipose tissue inflammation: novel insight into the role of macrophages and lymphocytes. Curr Opin Clin Nutr Metab Care.

[93] Sanz Y, Moya-Pérez A (2014). Microbiota, inflammation and obesity. Adv Exp Med Biol.

[94] Bleau C, Karelis AD, St-Pierre DH, Lamontagne L (2015). Crosstalk between intestinal microbiota, adipose tissue and skeletal muscle as an early event in systemic low grade inflammation and the development of obesity and diabetes. Diabetes Metab Res Rev.

[95] Gangarapu V, Yıldız K, Ince AT, Baysal B (2014). Role of gut microbiota: obesity and NAFLD. Turk J Gastroenterol.

[96] Moran CP, Shanahan F (2014). Gut microbiota and obesity: role in aetiology and potential therapeutic target. Best Pract Res Clin Gastroenterol.

[97] Remely M, Aumueller E, Jahn D, Hippe B, Brath H, Haslberger AG (2014). Microbiota and epigenetic regulation of inflammatory mediators in type 2 diabetes and obesity. Benef Microbes.

[98] Shen J, Obin MS, Zhao L (2013). The gut microbiota, obesity and insulin resistance. Mol Aspects Med.

[99] Kim KA, Gu W, Lee IA, Joh EH, Kim DH (2012). High fat diet-induced gut microbiota exacerbates inflammation and obesity in mice via the TLR4 signaling pathway. PLoS ONE.

[100] Tsukumo DM, Carvalho-Filho MA, Carvalheira JB, Prada PO, Hirabara SM, Schenka AA, Araújo EP, Vassallo J, Curi R, Velloso LA, Saad MJ (2007). Loss-of-function mutation in Toll-like receptor 4 prevents diet-induced obesity and insulin resistance. Diabetes.

[101] Song MJ, Kim KH, Yoon JM, Kim JB (2006). Activation of toll-like receptor 4 is associated with insulin resistance in adipocytes. Biochem Biophys Res Commun.

[102] Michelsen KS, Wong MH, Shah PK, Zhang W, Yano J, Doherty TM, Akira S, Rajavashisth TB, Arditi M (2004). Lack of toll-like receptor 4 or myeloid differentiation factor 88 reduces atherosclerosis and alters plaque phenotype in mice deficient in apolipoprotein E. Proc Natl Acad Sci U S A.

[103] Magouliotis DE, Tasiopoulou VS, Sioka E, Chatedaki C, Zacharoulis D (2017). Impact of bariatric surgery on metabolic and gut microbiota profile: a systematic review and meta-analysis. Obes Surg.

[104] Guo Y, Huang ZP, Liu CQ, Qi L, Sheng Y, Zou DJ (2018). Modulation of the gut microbiome: a systematic review of the effect of bariatric surgery. Eur J Endocrinol.

[105] Zhang H, DiBaise JK, Zuccolo A, Kudrna D, Braidotti M, Yu Y, Parameswaran P, Crowell MD, Wing R, Rittmann BE, Krajmalnik-Brown R (2009). Human gut microbiota in obesity and after gastric bypass. Proc Natl Acad Sci U S A.

[106] Furet JP, Kong LC, Tap J, Poitou C, Basdevant A, Bouillot JL, Mariat D, Corthier G, Doré J, Henegar C, Rizkalla S, Clément K (2010). Differential adaptation of human gut microbiota to bariatric surgery- induced weight loss: links with metabolic and low- grade inflammation markers. Diabetes.

[107] Palleja A, Kashani A, Allin KH, Nielsen T, Zhang C, Li Y, Brach T, Liang S, Feng Q, Jørgensen NB, Bojsen-Møller KN, Dirksen C, Burgdorf KS, Holst JJ, Madsbad S, Wang J, Pedersen O, Hansen T, Arumugam M (2016). Roux-en-Y gastric bypass surgery of morbidly obese patients induces swift and persistent changes of the individual gut microbiota. Genome Med.

[108] Tremaroli V, Karlsson F, Werling M, Ståhlman M, Kovatcheva-Datchary P, Olbers T, Fändriks L, le Roux CW, Nielsen J, Bäckhed F (2015). Roux- en-Y gastric bypass and vertical banded gastroplasty induce long-term changes on the human gut microbiome contributing to fat mass regulation. Cell Metab.

[109] Debédat J, Clément K, Aron-Wisnewsky J (2019). Gut microbiota dysbiosis in human obesity: impact of bariatric surgery. Curr Obes Rep.

[110] Aron-Wisnewsky J, Doré J, Clement K (2012). The importance of the gut microbiota after bariatric surgery. Nat Rev Gastroenterol Hepatol.

[111] Furet JP (2010). Differential adaptation of human gut microbiota to bariatric surgery induced weight loss: links with metabolic and low-grade inflammation markers. Diabetes.

[112] Murphy R (2017). Differential changes in gut microbiota after gastric bypass and sleeve gastrectomy bariatric surgery vary according to diabetes remission. Obes Surg.

[113] Li JV, Ashrafian H, Bueter M, Kinross J, Sands C, le Roux CW, Bloom SR, Darzi A, Athanasiou T, Marchesi JR, Nicholson JK, Holmes E (2011). Metabolic surgery profoundly influences gut microbial-host metabolic cross-talk. Gut.

[114] Wong VW, Chitturi S, Wong GL, Yu J, Chan HL, Farrell GC (2016). Pathogenesis and novel treatment options for non-alcoholic steatohepatitis. Lancet Gastroenterol Hepatol.

[115] Pournaras DJ, Glicksman C, Vincent RP, Kuganolipava S, Alaghband-Zadeh J, Mahon D, Bekker JHR, Ghatei MA, Bloom SR, Walters JRF (2012). The role of bile after Roux-en-Y gastric bypass in promoting weight loss and improving glycaemic control. Endocrinol.

[116] Seyfried F, Phetcharaburanin J, Glymenaki M, Nordbeck A, Hankir M, Nicholson JK, Holmes E, Marchesi JR, Li JV (2021). Roux-en-Y gastric bypass surgery in Zucker rats induces bacterial and systemic metabolic changes independent of caloric restriction-induced weight loss. Gut Microbes.

[117] Koulas SG, Stefanou CK, Stefanou SK, Tepelenis K, Zikos N, Tepetes K, Kapsoritakis A (2021). Gut microbiota in patients with morbid obesity before and after bariatric surgery: a ten-year review study (2009–2019). Obes Surg.

[118] Ilhan Z, DiBaise JK, Dautel SE, Isern NG, Kim Y, Hoyt DW, Schepmoes AA, Brewer HM, Weitz KK, Metz TO (2020). Temporospatial shifts in the human gut microbiome and metabolome after gastric bypass surgery. NPJ Biofilms Microbiomes.

[119] Talavera-Urquijo E, Beisani M, Balibrea JM, Alverdy JC (2020) Is bariatric surgery resolving NAFLD via microbiota-mediated bile acid ratio reversal? A comprehensive review. Surg Obes Relat Dis 16:1361–1369 10.1016/j.soard.2020.03.013 32336663

[120] Williams CD, Stengel J, Asike MI, Torres DM, Shaw J, Contreras M, Landt CL, Harrison SA (2011). Prevalence of nonalcoholic fatty liver disease and non-alcoholic steatohepatitis among a largely middle-aged population utilizing ultrasound and liver biopsy: a prospective study. Gastroenterol.

[121] Paquissi FC (2016). Immune imbalances in non-alcoholic fatty liver disease: from general biomarkers and neutrophils to interleukin-17 axis activation and new therapeutic targets. Front Immunol.

[122] Hazlehurst JM, Woods C, Marjot T, Cobbold JF, Tomilnson JW (2016). Non-alcoholic fatty liver disease and diabetes. Metab.

[123] Forlani G, Giorda C, Manti R, Mazzella N, De Cosmo S, Rossi MC, Nicolucci A, Di Bartolo P, Ceriello A, Guida P; AMD-Annals Study Group (2016). The burden of NAFLD and its characteristics in a nationwide population with type 2 diabetes. J Diabetes Res.

[124] Calle EE, Rodriguez C, Walker- Thurmond K, Thun MJ (2003). Overweight, obesity, and mortality from cancer in a prospectively studied cohort of U.S. adults. N Engl J Med.

[125] Aron-Wisnewsky J, Vigliotti C, Witjes J, Le P, Holleboom AG, Verheij J, Nieuwdorp M, Clément K (2020). Gut microbiota and human NAFLD: disentangling microbial signatures from metabolic disorders. Nat Rev Gastroenterol Hepatol.

[126] Chen J, Vitetta L (2020). Letter to the Editor: Could butyrate be incorporated with Farnesoid X receptor agonist cilofexor to enhance primary sclerosing cholangitis treatment?. Hepatol.

[127] Chen J, Vitetta L (2020). Butyrate in inflammatory bowel disease therapy. Gastroenterol.

[128] Chen J, Vitetta L (2020). Gut microbiota metabolites in NAFLD pathogenesis and therapeutic implications. Int J Mol Sci.

[129] Sohail MU, Althani A, Anwar H, Rizzi R, Marei HE (2017). Role of the gastrointestinal tract microbiome in the pathophysiology of diabetes mellitus. J Diabetes Res.

[130] Jasirwan COM, Muradi A, Hasan I, Simadibrata M, Rinaldi I (2021). Correlation of gut Firmicutes/Bacteroidetes ratio with fibrosis and steatosis stratified by body mass index in patients with non-alcoholic fatty liver disease. Biosci Microbiota Food Health.

[131] Nseir W, Artul S, Nasrallah N, Mahamid M (2016). The association between primary bacteremia of presumed gastrointestinal origin and nonalcoholic fatty liver disease. Dig Liver Dis.

[132] Kolodziejczyk AA, Zheng D, Shibolet O, Elinav E (2019) The role of the microbiome in NAFLD and NASH. EMBO Mol Med 11:e9302 10.15252/emmm.201809302 PMC6365925 30591521

[133] Chen J, Thomsen M, Vitetta L (2019). Interaction of gut microbiota with dysregulation of bile acids in the pathogenesis of nonalcoholic fatty liver disease and potential therapeutic implications of probiotics. J Cell Biochem.

[134] Lassailly G, Caiazzo R, Buob D, Pigeyre M, Verkindt H, Labreuche J, Raverdy V, Leteurtre E, Dharancy S, Louvet A, Romon M, Duhamel A, Pattou F, Mathurin P (2015). Bariatric surgery reduces features of nonalcoholic steatohepatitis in morbidly obese patients. Gastroenterol.

[135] Bell LN, Temm CJ, Saxena R, Vuppalanchi R, Schauer P, Rabinovitz M, Krasinskas A, Chalasani N, Mattar SG (2010). Bariatric surgery- induced weight loss reduces hepatic lipid peroxidation levels and affects hepatic cytochrome P-450 protein content. Ann Surg.

[136] Karcz WK, Krawczykowski D, Kuesters S, Marjanovic G, Kulemann B, Grobe H, Karcz-Socha I, Hopt UT, Bukhari W, Grueneberger JM (2011). Influence of sleeve gastrectomy on NASH and type 2 diabetes mellitus. J Obes.

[137] Billeter AT, Senft J, Gotthardt D, Knefeli P, Nickel F, Schulte T, Fischer L, Nawroth PP, Büchler MW, Müller-Stich BP (2016). Combined non-alcoholic fatty liver disease and type 2 diabetes mellitus: sleeve gastrectomy or gastric bypass? A controlled matched pair study of 34 patients. Obes Surg.

[138] Algooneh A, Almazeedi S, Al-Sabah S, Ahmed M, Othman F (2016). Nonalcoholic fatty liver disease resolution following sleeve gastrectomy. Surg Endosc.

[139] Praveen Raj P, Gomes RM, Kumar S, Senthilnathan P, Karthikeyan P, Shankar A, Palanivelu C (2015). The effect of surgically induced weight loss on nonalcoholic fatty liver disease in morbidly obese Indians: “NASHOST” prospective observational trial. Surg Obes Relat Dis.

[140] Clark JM, Alkhuraishi AR, Solga SF, Alli P, Diehl AM, Magnuson TH (2005). Roux-en-Y gastric bypass improves liver histology in patients with non-alcoholic fatty liver disease. Obes Res.

[141] Barker KB, Palekar NA, Bowers SP, Goldberg JE, Pulcini JP, Harrison SA (2006). Non-alcoholic steatohepatitis: effect of Roux-en-Y gastric bypass surgery. Am J Gastroenterol.

[142] Liu X, Lazenby AJ, Clements RH, Jhala N, Abrams GA (2007). Resolution of nonalcoholic steatohepatitis after gastric bypass surgery. Obes Surg.

[143] Furuya CK, de Oliveira CP, de Mello ES, Faintuch J, Raskovski A, Matsuda M, Vezozzo DC, Halpern A, Garrido AB, Alves VA (2007). Effects of bariatric surgery on nonalcoholic fatty liver disease: preliminary findings after 2 years. J Gastroenterol Hepatol.

[144] Froylich D, Corcelles R, Daigle C, Boules M, Brethauer S, Schauer P (2016). Effect of Roux-en-Y gastric bypass and sleeve gastrectomy on nonalcoholic fatty liver disease: a comparative study. Surg Obes Relat Dis.

[145] Kalinowski P, Paluszkiewicz R, Wróblewski T, Remiszewski P, Grodzicki M, Bartoszewicz Z, Krawczyk M (2017). Ghrelin, leptin, and glycemic control after sleeve gastrectomy versus Roux-en-Y gastric bypass—results of a randomized clinical trial. Surg Obes Relat Dis.

[146] Le Roux CW, Aylwin SJ, Batterham RL, Borg CM, Coyle F, Prasad V, Shurey S, Ghatei MA, Patel AG, Bloom SR (2006). Gut hormone profiles following bariatric surgery favor an anorectic state, facilitate weight loss, and improve metabolic parameters. Ann Surg.

[147] Musso G, Gambino R, Cassader M (2011). Interactions between gut microbiota and host metabolism predisposing to obesity and diabetes. Annu Rev Med.

[148] Cordeiro L, Campos JM, de Paula PS, Vilar L, Lopes E, de Arruda PC, Ramos A, Ferraz Á (2013). Nonalcoholic steatohepatitis on preoperative period of gastric bypass: lack of correlation with degree of obesity. ABCD Arq Bras Cir Dig.

